# Mating competitiveness of sterile male *Anopheles coluzzii* in large cages

**DOI:** 10.1186/1475-2875-13-460

**Published:** 2014-11-26

**Authors:** Hamidou Maïga, David Damiens, Abdoulaye Niang, Simon P Sawadogo, Omnia Fatherhaman, Rosemary S Lees, Olivier Roux, Roch K Dabiré, Georges A Ouédraogo, Fréderic Tripet, Abdoulaye Diabaté, Jeremie RL Gilles

**Affiliations:** Institut de Recherche en Sciences de la Santé/Centre Muraz, BP 545, Bobo-Dioulasso, Burkina Faso; Insect Pest Control Laboratory, Joint FAO/IAEA Division of Nuclear Techniques in Food and Agriculture, International Atomic Energy Agency, Wagramerstraße 5, PO Box 100, A-1400 Vienna, Austria; Keele University, Staffordshire, ST5 5BG UK; Epidemiology Department, Tropical Medicine Research Institute, PO Box 1304, Khartoum, Sudan; Polo d’Innovazione Genomica, Genetica e Biologia, Polo Unico di Medicina Santa Maria della Misericordia, Perugia, Italy; Institut de Recherche pour le Développement, MIVEGEC (IRD 224-CNRS 5290-UM1-UM2), 911 Avenue Agropolis, BP 64501, 34394 Montpellier, Cedex 5, France; Université Polytechnique de Bobo-Dioulasso, 01 BP 1091, Bobo-01, Burkina Faso

**Keywords:** Male mating biology, *Anopheles coluzzii*, Sterile insect technique, Competitiveness

## Abstract

**Background:**

Understanding the factors that account for male mating competitiveness is critical to the development of the sterile insect technique (SIT). Here, the effects of partial sterilization with 90 Gy of radiation on sexual competitiveness of *Anopheles coluzzii* allowed to mate in different ratios of sterile to untreated males have been assessed. Moreover, competitiveness was compared between males allowed one *versus* two days of contact with females.

**Methods:**

Sterile and untreated males four to six days of age were released in large cages (~1.75 sq m) with females of similar age at the following ratios of sterile males: untreated males: untreated virgin females: 100:100:100, 300:100:100, 500:100:100 (three replicates of each) and left for two days. Competitiveness was determined by assessing the egg hatch rate and the insemination rate, determined by dissecting recaptured females. An additional experiment was conducted with a ratio of 500:100:100 and a mating period of either one or two days. Two controls of 0:100:100 (untreated control) and 100:0:100 (sterile control) were used in each experiment.

**Results:**

When males and females consort for two days with different ratios, a significant difference in insemination rate was observed between ratio treatments. The competitiveness index (C) of sterile males compared to controls was 0.53. The number of days of exposure to mates significantly increased the insemination rate, as did the increased number of males present in the untreated: sterile male ratio treatments, but the number of days of exposure did not have any effect on the hatch rate.

**Discussion:**

The comparability of the hatch rates between experiments suggest that *An. coluzzii* mating competitiveness experiments in large cages could be run for one instead of two days, shortening the required length of the experiment. Sterilized males were half as competitive as untreated males, but an effective release ratio of at least five sterile for one untreated male has the potential to impact the fertility of a wild female population. However, further trials in field conditions with wild males and females should be undertaken to estimate the ratio of sterile males to wild males required to produce an effect on wild populations.

## Background

Malaria remains a serious threat to world health, causing more than 600,000 deaths each year in endemic zones mainly in children under five[1]. Current malaria control strategies are mainly based on the use of insecticides, in the form of long-lasting insecticidal nets (LLINs) and indoor residual spraying (IRS), and on the treatment of infected people with artemisinin-combination therapy (ACT)[1]. However, the emergence and rapid spread of both vector resistance to common insecticide classes and *Plasmodium* resistance to available anti-malarial drugs, such as chloroquine and sulphadoxine–pyrimethamine, undermine current malaria control strategies that have reduced the malaria burden in many endemic areas. In several countries in West Africa, resistance to pyrethroids[2–4], the only insecticide class approved for impregnation of bed nets[5], could impact current malaria control[6]. Thus, there is growing concern that malaria elimination will not be achieved without the introduction of novel control tools, and research into alternative strategies against adult vectors has been increasing[7].

A renewed interest in the development of environment-friendly vector control strategies using sterile insects raises hopes of being able to reduce the high reproductive rate of mosquitoes. The sterile insect technique (SIT) is based on inundative and repeated releases of sterile insects to induce sterility in the wild population and consequently suppress the target pest species[8]. The SIT has been successfully used against a variety of pests and vectors, including new world screwworm[9], *Ceratitis capitata*[10] and *Glossina austeni* in Zanzibar[11]. In El Salvador, experimental releases of chemosterilized male *Anopheles albimanus* drastically reduced the wild mosquito population in a pilot area[12]. However, several trials conducted in the 1970s produced limited success due to the poor competitiveness of released males for wild females (reviewed in[13]). Even in the laboratory, sterile male *Anopheles quadrimaculatus* were unable to control a caged field population[14].

The success of an area-wide integrated vector management (AW-IPM) programme that includes an SIT component clearly depends on the ability of laboratory mass-produced and sterilized males to locate and compete for mates in the wild to transfer their sterile sperm[15]. Colonization and mass-rearing may modify reproductive ability and alter the quality of males produced[16]. Furthermore, sterilization by irradiation induces damage in the somatic cells of males, in a dose-dependent manner depending on the age of pupae at the time of irradiation[17, 18], and lead to reduced mating competitiveness[18, 19].

Studies into the mating competitiveness of sterile versus untreated *Anopheles coluzzii,* one of the major vectors of malaria in sub-Saharan Africa, have mainly been carried out in small cage laboratory settings[17] and thus may not accurately reflect performance in the natural environment[20]. A recent study reported the first comparisons of survival and mating success of a laboratory strain of the Mopti form of *Anopheles gambiae sensu stricto versus* wild individuals in semi-field conditions[21]. The same study showed how laboratory rearing was affecting the mating behaviour of *An. gambiae s.s.* resulting in lower survival and mating success than field progeny reared outdoors. In *Aedes albopictus*, one study has shown that the competitiveness of sterilized males was similar when assessed in small laboratory cages or in field cages[22]. However, other studies have shown that colonized strains could develop different swarming and mating behaviour than wild populations[16, 23] and thus could fail to locate mates in the wild. This suggests that trials in large field cages are required in order to evaluate the effectiveness and reliability of sterile males in settings that include natural environmental variations before releasing mosquitoes into the open field.

In this study, the effect of partial sterilization with 90 Gy irradiation[24] on sexual competitiveness of *Anopheles coluzzii* in three different ratios of a sterile to untreated males in large cages has been assessed. The competitiveness of sterile males after one and after two days of contact between males and females was measured to determine the dynamics of this competition in large cages.

## Methods

### Mosquito strain

The strain of *An. coluzzii* (formerly *An. gambiae* M molecular form) used in this study was obtained from the Institut de Recherche en Sciences de la Santé (IRSS) in Bobo-Dioulasso, Burkina Faso. The strain was established in August 2008 from gravid females collected in Bama, Vallée du Kou (11°23′N, 4°24′W). The colony was transferred to the Insect Pest Control Laboratory (IPCL), FAO/IAEA Joint Division, Seibersdorf, Austria in January 2013. Mosquitoes were reared at a density of 200 larvae per tray (30 × 40 cm) with 1 L of deionized water, and fed with the IAEA larval diet (Tuna Meal: 5 g/L; Bovine Liver Powder: 5 g/L; Vitamin Mix: 4.6 g/L)[25] at 0.02 ml/larvae/day for the first two days; then 0.04 ml/larvae/day on the third and fourth days, and 0.08 ml/larvae/day until pupation. The colony was maintained in climate-controlled insectaries (27 ± 1°C, 80 ± 10% RH and 12 L: 12D with one hour simulated dawn and dusk). Adults were maintained in 30 × 30 × 30 cm cages with 5% sucrose solution with methylparaben[26] provided *ad libitum*. Frozen blood was used for rearing and for experimental females prior to oviposition. Females were allowed to oviposit on wet filter paper. Eggs were conserved by storing folded filter papers in petri dishes sealed with parafilm for two to four days in laboratory conditions (27 ± 1°C, 80 ± 10% RH), to allow the synchronization of hatching.

### Collection and irradiation of pupae

Individuals which pupated between 09.00 and 15.00 hours each day were collected and irradiated at 11.00 the following day, so that pupal age was between 22 and 26 hours at the time of irradiation with 90 Gy in a Gamma Cell 220 ^60^Co self-contained gamma source (dose rate 12 Gy/min)[18]. Prior to the sterilization, pupae were separated by sex under a microscope. Batches of 100 to 200 pupae were irradiated at a time. A dosimetry system based on the Gafchromic® HD-810 film (International Specialty Products, NJ, USA) was used to measure the dose accuracy after irradiation.

### Assaying competitiveness of irradiated *Anopheles coluzzii*males

Experiments were conducted in large cages (~1.75 sq m, Live Monarch, Boca Raton, USA) at the FAO/IAEA Insect Pest Control Laboratory climate-controlled greenhouse in Seibersdorf (Austria) under natural light, average temperatures of 24.7 ± 0.6°C in the morning and 26.7 ± 0.2°C in the evening, and 50% relative humidity. One tray (30 × 40 cm) containing 1 L tap water was provided as resting site in each cage with two containers of 5% sugar solution provided nearby.

Four to six day-old sterilized and untreated males were released into large cages where they were allowed to compete for virgin females of the same age for two nights in the following ratios (sterile males: untreated males: virgin females): 100:100:100, 300:100:100 and 500:100:100, three replicates per ratio. In addition, ratios of 0:100:100 (untreated control) and 100:0:100 (sterile control) were included in each of the three replicates. In each replicate, females were added few minutes after the cages were filled at 11.00 with males.

On the third day following release, all the females were removed from the cages and put in 30 × 30 × 30 cm cages for blood feeding for 30 min on each of 2 consecutive days on fresh bovine blood. All blood-fed females were kept for three days after the last blood meal for egging *en masse*. The females were allowed to lay eggs for two consecutive days. Each day, newly laid eggs were collected, rinsed and allowed to hatch over two days. The sterility rate was then assessed by counting the number of hatched eggs under a stereomicroscope. To assess the insemination state of females, spermathecae were dissected under a stereomicroscope and the presence/absence of spermatozoa was observed at 400X magnification.

### Analysing competition depending on time of contact

The temporal dynamics of competition were assessed under the same conditions as above using only the 500:100:100 ratio of sterile males: untreated males: virgin females. The insemination and the hatch rates of females was measured as previously after one and two days and compared. Three replicates were carried out, including with sterile and untreated controls each time.

### Statistical analysis

In the competitiveness experiment, the analyses of hatch rate and insemination rate were carried out using the generalized linear mixed model (GLMM) procedure with binomial error and logit link (‘glmer’ function in the ‘lme4’ package). Each egg was coded as hatched or unhatched (binary) and females as inseminated or not inseminated. The different male ratios were used as fixed effect and the cage identity (ratio × replicate) was assigned as random effect. Pair-wise comparisons between the different male ratios were performed with a Tukey’s *posthoc* test (‘glht’ function in the ‘multcomp’ package). The same GLMM procedure was adopted for the experiment on dynamics of competition. The number of days (one or two), the male ratio and their interaction were used as fixed effects and the cage identity was assigned as random effect. For model selection, the stepwise removal of terms has been used, followed by likelihood ratio tests (LRT). Term removals that significantly reduced explanatory power (*P* <0.05) were retained in the minimal adequate model. All statistical analyses and graphics were performed using R 2.15.2 and GraphPad Prism v.5.0 software, respectively.

The formula ((Hn-Ho) / (Ho-Hs))*(N/S) was used to calculate the Fried competitiveness index (C) for the ratio 100:100:100 as described by Fried,[27], where Hn and Hs denotes hatch rate from eggs of females mated with untreated and sterile males, respectively, Ho is the observed egg hatch rate and N is the number of untreated males and S is the number of sterile males.

## Results

### Competitiveness of irradiated *Anopheles coluzzii*males

When males and females consorted for two days, a significant difference in insemination rate was observed with different ratios of sterile to untreated males (LRT *χ*^2^ = 35.86; *df =* 4; *P <* 0.001) (Table 1). The insemination rate in all ratios was significantly different from both the sterile and the untreated controls (Tukey’s *posthoc* test: all *P <*0.01). The insemination rate of females in the untreated males control was also significantly different from the rate in the sterile control (Tukey’s *posthoc* test: *Z =* -3.47; *P =* 0.004). However, insemination rate in the three ratios tested was not significantly different from each other (Tukey’s *posthoc* test: all *P* >0.05).

**Table 1:** **Competitiveness index (C) of sterile** ***Anopheles coluzzii*** **males in large cages, measured with different ratios of sterile to untreated males**

Ratio	Insemination rate (%)	S /N	Hatch rate (%)	Competitiveness index (C)
Fertile control	81.28 ± 3.11^a^		86.47 (Hn)	
Sterile control	67.38 ± 1.55^b^		18.64 (Hs)	
100:100:100	92.23 ± 1.28^c^	1	63.99 (Ho)	0.53 ± 0.16
300:100:100	94.83 ± 0.41^c^	3	55.24 (Ho)	
500:100:100	93.89 ± 1^c^	5	37 (Ho)	

Insemination rate ± SE (%) of females as a function of the different ratios (sterile males: untreated males: virgin untreated females). Different letters (a, b, c) indicate significant differences between the ratios (Tukey’s posthoc test *P <* 0.05). Hn and Hs are the hatch rate from eggs of females mated with untreated (untreated control) or sterile (sterile control) males respectively. Ho is the observed egg hatch rate for each ratio and N and S as the numbers of untreated and sterile males respectively.

**Figure 1:**
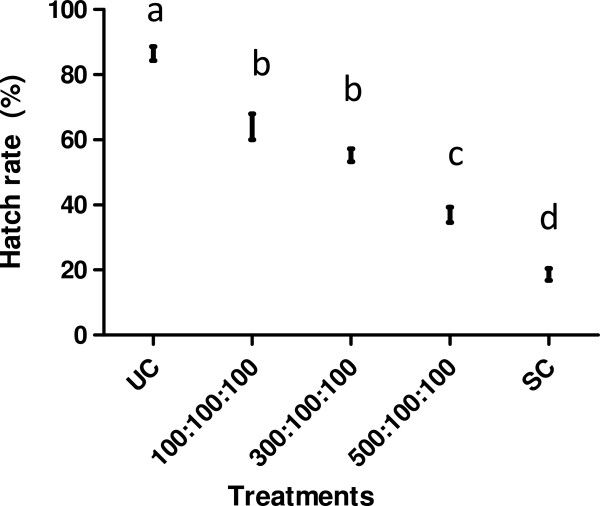
**Hatch rate (%) as a function of different ratios (sterile males: untreated males: virgin females).** UC = untreated control; SC = sterile control. Different letters indicate significant differences between the ratios (Tukey’s *posthoc* test, P <0.05).

The egg hatch rate was significantly influenced by the ratio of sterilized males (LRT *χ*^2^ = 52.50; df = 4, *P <* 0.001) in the competitiveness experiment (Figure 1). All ratios were significantly different from both the sterile and the untreated controls (Tukey’s *posthoc* test: all *P <*0.001) and all ratios were significantly different from each other (Tukey’s *posthoc* test: all *P <*0.001), except the ratios 100:100:100 and 300:100:100 (Tukey’s *posthoc* test: *Z =* -2.29; *P =* 0.14; Figure 1).

The competitiveness index (C) of sterilized males compared to controls was 0.53 meaning that sterile males were about half as competitive as untreated males (Table 1).

### Effect of time of contact between males and females

Both the number of days of exposure to mates and the sterile: untreated male ratio had a significant effect on the insemination rate (LRT χ^2^ = 16.66; df = 1, *P <* 0.001 and LRT χ^2^ = 19.53; df = 2, *P <* 0.001 respectively; Table 2). Insemination rate was significantly higher with the ratio 500:100:100 compared to both sterile and untreated controls (Tukey’s *posthoc* test: Z = 5.03; *P <*0.001 and Z = 4.45; *P <*0.001 respectively; Table 2), in accordance with the results of the first experiment.

**Table 2:** **Effect of time of contact between males and females on insemination and hatch rates**

	Sterile control			500:100 :100			Untreated control		
Duration of contact	1 day	2 days		1 day	2 days		1 day	2 days	
IR ± SE	59.23 ± 10.97	82.58 ± 5.04	*	85.25 ± 8.90	92.08 ± 1.49	NS	64.10 ± 10.63	82.87 ± 2.59	*
HR ± SE	27.24 ± 6.39	24.68 ± 4.82	NS	43.37 ± 6.02	38.40 ± 6.45	NS	90.07 ± 1.61	88.09 ± 3.18	NS

The insemination rate (IR) and the hatch rate (HR) were compared over a time of one or two days of contact between males and females mosquitoes. SE = Standard Error .NS non-significant and *P < 0.05 between days.

The number of days of exposition did not have any effect of the hatch rate (LRT χ^2^ = 2.82; df = 1; *P =* 0.09; Table 2).

## Discussion

Irradiation for sterilization had an impact on the ability of *An. coluzzii* males to compete for mates in a large cage setting. The Fried competitiveness index[27] was estimated from the experimental data and showed that the sterile males were half as competitive as the untreated males when allowed to mate over two nights. Numerous experiments have shown the decrease in male competitiveness due to irradiation[13, 17] and see Table 2 in[28], and most researchers have explained it as being due to a decrease in survival, mating ability or sperm stock[29–31]. However, the fact that frequently the differences in competitiveness are more visible in field cages and in field than in small cages[32], suggests that other parameters could be influenced by irradiation such as flight ability, or ability to detect or inseminate the female. In this study, insemination rate by sterile males was significantly lower compared to that of untreated males even in the controls when they were not in direct competition, suggesting that the males are less able to find females or achieve successful copulation if they do. Irradiation is known to reduce the walking speed of adult beetles[32], increase inactivity periods in fruit fly *Bactrocera tryoni*[33] and considerably modify the fine structure of the fibrillar flight muscles of *Musca domestica*[34]. In a situation of competition, such somatic damage could be very detrimental for sterile males. Indeed, it is known that males in swarms engage in intense competition to intercept females. Diabaté *et al.*[35] proposed that swarms of *An. gambiae* are lek-like, and incorporate characteristics of scramble-mating competition. Several models of mating pertaining to leks are described, and the hotshot model specifically states that males are not equal[35]. Successful individuals attract females and "poor quality males" gather around these successful ones to mate with incoming females. It is possible that in this scenario sterile males are the "poor quality males", achieving insemination of fewer females. Andreasen and Curtis[17] also noticed a fitness cost of sterilization to *Anopheles* species, indicating that some sterile males suffered a delay in swarm participation or a failure to initiate mating swarms. This incapacity to join a swarm could result in a reduced insemination rate even within the same cohort of mosquitoes. Similar observations have been made when sterile *Anopheles arabiensis* mosquitoes were allowed to mate for seven days in large cages compared to small cages[29]. This could explain the reason why the competitiveness of sterile males under large cage settings is important for any genetic control programme.

To overcome the decrease in competitiveness caused by irradiation, an over-flooding ratio of sterile males has been proposed when releasing sterile males for population suppression[36]. A reduction in the radiation dose applied would minimize the impact on competitiveness of males but also reduce the induced sterility; thus for suppression programmes a balance must be struck between level of induced sterility and compensatory release ratio[15, 17]. In this study, the insemination and hatch rates were significantly influenced by the ratio of sterile to untreated males meaning that an increase in number of sterile males could significantly impact on vector populations. Indeed, the ratio 500:100:100 showed a better sterilization of the population but still was unable to induce the level of sterility observed in the sterile control treatment, meaning that a release ratio greater than 5:1 would be required to achieve population eradication. These variations in competitiveness between ratios could be due to several factors including the differences in individual male sterility level. The sterility level has been shown to be related to pupal age at irradiation time and the dose received[29] for the same irradiation treatment. Reduction in mating competitiveness of males irradiated as pupae with a partially sterilizing dose could be overcome, according to Helinski *et al.*[29], by a three-fold increase in their number compared to un-irradiated males in large cages. Male *An. quadrimaculatus* sterilized by gamma irradiation were not as competitive as untreated males in the laboratory[37] but at high enough ratios of sterile to untreated males (6:1 and 10:1) the hatch rate decreased more than 80%. Other studies have stated that for full irradiation, the level of irradiation needs to be higher[17]. However, the higher is the dose, the greater the impact on competitiveness and thus, the higher flooding ratio of sterile males[15] that will be needed to achieve the same impact on a wild population. A field competitiveness test with the MACHO strain gave a competitiveness index of between 0.78 and 0.80[38]. More recently, Yamada *et al.*[19] found in a mating competitiveness study of *An. arabiensis* sterilized with 75 Gy, that a 10:1 ratio of sterile to untreated males produced 81% induced sterility in the female cage population. The same study calculated a competitiveness index of around 0.53 for 5:1 and 10:1 ratios.

In *An. coluzzii*, the number of days of contact between males and females did not influence the egg hatch rate in the current experimental design, suggesting that the proportion of sterile and untreated males that mated on the first and the second night did not change when a high ratio of sterile to untreated male were competing. In the 500:100:100 treatment, the insemination rate did not change according to the time of contact. Therefore, on a practical level this finding will allow researchers to save time since one day is enough to run a competitiveness test in large cages; the mortality will be lower and the experiment quicker to perform.

For SIT the cost of sterilization should be either compensated through over-flooding release ratios or minimized by reducing the irradiation dose, in order to maximize the efficiency of the technique[39]. Regardless, further trials in field conditions with wild or more recently colonized males should be undertaken in order to estimate the ratio of sterile males to wild males required to produce an effect on wild populations. Labelling the males with stable isotopes in order to determine clearly the origin of sperm transferred to inseminated females could be used to determinate when a female has mated with an untreated or a sterile male, and so the relative mating success after different lengths of time could be investigated.

## References

[1] WHO (2013). World Malaria Report 2013.

[2] Dabiré KR, Diabaté A, Namountougou M, Djogbenou L, Wondji C, Chandre F, Simard F, Ouédraogo JB, Martin T, Weill M, Baldet T, Perveen F (2012). Trends in Insecticide Resistance in Natural Populations of Malaria Vectors in Burkina Faso, West Africa: 10 Years’ Surveys. Insecticides-Pest Engineering.

[3] Namountougou M, Simard F, Baldet T, Diabaté A, Ouédraogo JB, Martin T, Dabiré KR (2012). Multiple insecticide resistance in Anopheles gambiae s.l. populations from Burkina Faso, West Africa. PLoS One.

[4] Ranson H, Abdallah H, Badolo A, Guelbeogo WM, Kerah-Hinzoumbe C, Yangalbe-Kalnone E, Sagnon N, Simard F, Coetzee M (2009). Insecticide resistance in *Anopheles gambiae*: data from the first year of a multi-country study highlight the extent of the problem. Malar J.

[5] Zaim M, Aitio A, Nakashima N (2000). Safety of pyrethroid-treated mosquito nets. Med Vet Entomol.

[6] Ranson H, N’Guessan R, Lines J, Moiroux N, Nkuni Z, Corbel V (2011). Pyrethroid resistance in African anopheline mosquitoes: what are the implications for malaria control?. Trends Parasitol.

[7] Vreysen MJ, Robinson AS, Hendrichs J (2007). Area-Wide Control of Insect Pests: From Research to Field Implementation.

[8] Knipling EF, Laven H, Craig GB, Pal R, Smith CN, Brown AWA (1968). Genetic control of insects of public health importance. Bull World Health Organ.

[9] Lindquist DA, Abusowa M, Klassen W, IAEA/FAO (1993). Eradication of the new World Screwworm from the Libyan Arab Jamahiriya. Management of Insect Pests: Nuclear and Related Molecular and Genetic Techniques.

[10] Vargas RI, Whitehand L, Walsh WA, Spencer JP, Hsu C, Hsu CL (1995). Aerial releases of sterile Mediterranean fruit fly (Diptera: Tephritidae) by helicopter: dispersal, recovery, and population suppression. J Econ Entomol.

[11] Vreysen MJB, Saleh KM, Ali MY, Abdulla AM, Zhu Z-R, Juma KG, Dyck VA, Msangi AR, Mkonyi PA, Feldmann HU (2000). *Glossina austeni* (Diptera: Glossinidae) eradicated on the island of Unguja, Zanzibar, using the sterile insect technique. J Econ Entomol.

[12] Dame DA, Lofgren CS, Ford HR, Boston MD, Baldwin KF, Jeffery GM (1974). Release of chemosterilized males for the control of *Anopheles albimanus* in El Salvador II. Methods of rearing, sterilization, and distribution. Am J Trop Med Hyg.

[13] Benedict MQ, Robinson AS (2003). The first releases of transgenic mosquitoes: an argument for the sterile insect technique. Trends Parasitol.

[14] Dame DA, Woodard DB, Ford HR, Weidhaas DE (1964). Field behavior of sexually sterile *Anopheles quadrimaculatus* males. Mosq News.

[15] Knipling EF (1955). Possibilities of insect control or eradication through the use of sexually sterile males. J Econ Entomol.

[16] Benedict MQ, Knols BGJ, Bossin HC, Howell PI, Mialhe E, Caceres C, Robinson AS (2009). Colonisation and mass rearing: learning from others. Malar J.

[17] Andreasen MH, Curtis CF (2005). Optimal life stage for radiation sterilization of *Anopheles* males and their fitness for release. Med Vet Entomol.

[18] Helinski MEH, Knols BG (2009). The influence of late-stage pupal irradiation and increased irradiated: un-irradiated male ratio of mating competitiveness of the malaria mosquito *Anopheles arabiensis* Patton. Bull Entomol Res.

[19] Yamada H, Vreysen MJ, Gilles JR, Munhenga G, Damiens DD (2014). The effects of genetic manipulation, dieldrin treatment and irradiation on the mating competitiveness of male *Anopheles arabiensis* in field cages. Malar J.

[20] Reisen WK, Takken W, Scott TW (2003). Lessons from the Past: Historical Studies by the University of Maryland and the University of California, Berkeley. Ecological Aspects for Application of Genetically Modified Mosquitoes.

[21] Paton D, Touré M, Sacko A, Coulibaly MB, Traoré SF, Tripet F (2013). Genetic and Environmental factors associated with laboratory rearing affect survival and assortative mating but not overall mating success in *Anopheles gambiae* sensu stricto. PLoS One.

[22] Madakacherry O, Lees RS, Gilles JRL (2014). *Aedes albopictus* (Skuse) males in laboratory and semi-field cages: release ratios and mating competitiveness. Acta Trop.

[23] Marchand RP (1985). A new cage for observing mating behavior of wild *Anopheles gambiae* in the laboratory. J Am Mosq Control Assoc.

[24] Helinski MEH, Parker AG, Knols BGJ (2009). Radiation biology of mosquitoes. Malar J.

[25] Damiens D, Benedict MQ, Wille M, Gilles JRL (2012). An inexpensive and effective larval diet for *Anopheles arabiensis* (Diptera: Culicidae): Eat like a horse, a bird or a fish?. J Med Entomol.

[26] Benedict MQ, Hood-Nowotny RC, Howell PI, Wilkins EE (2009). Methylparaben in *Anopheles gambiae* s.l. sugar meals increases longevity and malaria oocyst abundance but is not a preferred diet. J Insect Physiol.

[27] Fried M (1971). Determination of sterile-insect competitiveness. J Econ Entomol.

[28] Helinski MEH, Parker AG, Knols BG (2006). Radiation-induced sterility for pupal and adult stages of the malaria mosquito *Anopheles arabiensis*. Malar J.

[29] Helinski MEH, Knols BGJ (2008). Mating competitiveness of male *Anopheles arabiensis* mosquitoes irradiated with a partially or fully sterilizing dose in small and large laboratory cages. J Med Entomol.

[30] Oliva CF, Maier MJ, Gilles J, Jacquet M, Lemperiere G, Quilici S, Vreysen MJ, Schooneman F, Chadee DD, Boyer S (2012). Effects of irradiation, presence of females, and sugar supply on the longevity of sterile males *Aedes albopictus* (Skuse) under semi-field conditions on Reunion Island. Acta Trop.

[31] Damiens D, Vreysen MJB, Gilles JRL (2013). *Anopheles arabiensis* sperm production after genetic manipulation, dieldrin treatment, and irradiation. J Med Entomol.

[32] Ignatowicz S, Wesolowska B, Zaedee IH (1994). Detection of Irradiated Insect Pests in Stored Products: Locomotor Activity of Irradiated Adult Beetles. Proceedings of the 6th International Working Conference on Stored-Product Protection, 17–23 April 1994.

[33] Weldon CW, Prenter J, Taylor PW (2010). Activity patterns of Queensland fruit flies *(Bactrocera tryoni*) are affected by both mass‒rearing and sterilization. Physiol Entomol.

[34] Bhakthan NMG, Nair KK (1972). Fine structural damage in the somatic tissues of gamma-irradiated house fly. 1. Flight muscles. Ann Entomol Soc Am.

[35] Diabaté A, Yaro AS, Dao A, Diallo M, Huestis DL, Lehmann T (2011). Spatial distribution and male mating success of *Anopheles gambiae* swarms. BMC Evol Biol.

[36] Dame DA, Curtis CF, Benedict MQ, Robinson AS, Knols BGJ (2009). Historical applications of induced sterilisation in field populations of mosquitoes. Malar J.

[37] Patterson RS, Lofgren CS, Boston MD (1968). The sterile male technique for control of mosquitoes: a field cage study with *Anopheles quadrimaculatus*. Florida Entomol.

[38] Kaiser PE, Bailey DL, Lowe RE (1981). Realease strategy evaluation of sterile males of *Anopheles albimanus* with competitive mating. Mosq News.

[39] Parker A, Mehta K (2007). Sterile insect technique: a model for dose optimization for improved sterile insect quality. Florida Entomol.

